# Schizophrenia Patients Demonstrate Both Inter-Voxel Level and Intra-Voxel Level White Matter Alterations

**DOI:** 10.1371/journal.pone.0162656

**Published:** 2016-09-12

**Authors:** Chuanjun Zhuo, Xiaolei Ma, Hongru Qu, Lina Wang, Feng Jia, Chunli Wang

**Affiliations:** 1 Tianjin Anding Hospital, Tianjin Mental Health Center, Tianjin, China; 2 Department of Radiology and Tianjin Key Laboratory of Functional Imaging, Tianjin Medical University General Hospital, Tianjin, 300052, China; 3 Tianjin Anning Hospital, Tianjin, 300300, China; 4 Wenzhou seventh people's Hospital, Wenzhou, 325000, China; Banner Alzheimer's Institute, UNITED STATES

## Abstract

Fractional anisotropy (FA) and mean diffusivity (MD) are the most frequently used metrics to investigate white matter impairments in mental disorders. However, these two metrics are derived from intra-voxel analyses and only reflect the diffusion properties solely within the voxel unit. Local diffusion homogeneity (LDH) is a newly developed inter-voxel metric which quantifies the local coherence of water molecule diffusion in a model-free manner. In this study, 94 schizophrenia patients and 91 sex- and age-matched healthy controls underwent diffusion tensor imaging (DTI) examinations. White matter integrity was assessed by FA, MD and LDH. Group differences in these metrics were compared using tract-based spatial statistics (TBSS). Compared with healthy controls, schizophrenia patients exhibited reduced FA and increased MD in the corpus callosum, cingulum, internal capsule, fornix and widespread superficial white matter in the frontal, parietal, occipital and temporal lobes. We also found decreased LDH in the corpus callosum, cingulum, internal capsule and fornix in schizophrenia. Our findings suggest that both intra-voxel and inter-voxel diffusion metrics are able to detect impairments in the anisotropic white matter regions, and intra-voxel diffusion metrics could detect additional impairments in the widespread isotropic white matter regions in schizophrenia.

## Introduction

Diffusion tensor imaging (DTI) is commonly used to investigate white matter (WM) alterations in the human brain [1,2]. Based on the diffusion tensor model, fractional anisotropy (FA) and mean diffusivity (MD) are the most frequently used metrics to characterize the diffusion of water molecules [3,4]. FA represents a normalized ratio of diffusion directionality, while MD quantifies the bulk mobility of water molecules; thus, these indices provide information on white matter integrity from different perspectives. Although FA and MD are the two most commonly used metrics for evaluating alterations in white matter integrity, they are intra-voxel diffusion metrics and only reflect diffusion properties solely within the voxel unit [3,5,6].

Several studies have reported that inter-voxel diffusion metrics are informative and useful [5–7]. These inter-voxel metrics are able to reveal significant region-specific white matter alterations in some neuropsychical diseases and provide useful information for computer-aided diagnosis, although inter-voxel and intra-voxel metrics have shown inconsistent findings [7–9].

In a prior study, a novel inter-voxel metric was proposed to quantify the local coherence of water molecule diffusion in a model-free manner, which is referred to as the local diffusion homogeneity (LDH) [9]. Because the profile of water molecule diffusion is related to the underlying white matter micro-structural properties, the LDH is supposed to reflect the local coherence of fiber orientations, myelination, diameter or density along each white matter tract. Some of these micro-structural factors cannot be interpreted by the traditional diffusion metrics, such as FA and MD. Therefore, LDH can serve as a complementary marker for investigating white matter alterations of the brain and can provide additional information for understanding the pathological mechanisms of neuropsychical diseases [9].

Many previous studies have confirmed that alterations of FA and MD can be found in multiple white matter regions in patients with schizophrenia [10–16]. Together, the findings suggest that impairments of white matter integrity are pathological features of schizophrenia. However, as mentioned above, all of these studies focused on the intra-voxel metrics. Therefore, using the inter-voxel metric to explore white matter alterations in patients with schizophrenia may provide additional information on the neural mechanisms of schizophrenia.

In the current study, we reconstructed the white matter skeleton of the brain using tract-based spatial statistics (TBSS) [17] and compared differences in the inter-voxel (LDH) and the intra-voxel (FA and MD) metrics within the skeleton between schizophrenia patients (n = 94) and healthy controls (n = 91). We hypothesized that both intra-voxel and inter-voxel metrics are able to detect white matter alterations in schizophrenia patients and that the distribution of abnormal white matter regions detected by the two types of metrics may be different.

## Methods

### Subjects

A total of 106 schizophrenia patients and 94 healthy subjects were enrolled in the present study. This study was approved by the Ethics Committee of Tianjin Medical University General Hospital, and written informed consent was obtained from each subject before study enrollment. Diagnosis for each schizophrenia patient was determined by the consensus of two clinical psychiatrists using the Structured Clinical Interview for DSM-IV (SCID). Inclusion criteria were age (16–60 years) and right-handedness. Exclusion criteria for all subjects were MRI contraindications, pregnancy, and histories of systemic medical illness, central nervous system disorder and head trauma, and substance abuse within the last 3 months or lifetime history of substance abuse or dependence. Additional exclusion criteria for healthy controls included a history of psychiatric disease and first-degree relatives with a psychotic disorder. After image quality assessment slice by slice by two professional radiologists, 12 patients and 3 controls were excluded due to poor quality of imaging data. The final sample included 94 schizophrenia patients and 91 healthy controls. Psychotic symptoms were quantified with the Positive and Negative Syndrome Scale (PANSS) [18].

### MRI Data Acquisition

MRI was performed using a 3.0-Tesla MR system (Discovery MR750, General Electric, Milwaukee, WI, USA). Tight but comfortable foam padding was used to minimize head motion, and earplugs were used to reduce scanner noise. DTI data were acquired by a spin-echo single-shot echo planar imaging (EPI) sequence with the following parameters: repetition time = 5800ms; echo time = 77ms; matrix = 128 × 128; field of view = 256 mm × 256 mm; in-plane resolution = 2 mm × 2 mm; slice thickness = 3mm without gap; 48 axial slices; b = 1000 s/mm^2^ and 25 encoding diffusion directions; 10 non-diffusion-weighted images (b = 0 s/mm^2^). The total acquisition time for DKI was 5 min and 54 s.

### Calculation of FA, MD and LDH

Eddy current-induced distortion and motion artifacts in the DTI dataset were corrected using affine alignment of each diffusion-weighted image to the b = 0 image. After skull-stripping, we calculated the diffusion tensor matrix for each subject in the native space. Next, three pairs of eigenvalues and eigenvectors were obtained from the diffusion tensor matrix. FA and MD images were calculated based on the three eigenvalues. We also calculated the LDH images (the pre-defined neighborhood was 27 voxels) according to a prior study [9]. All of the procedures described above were implemented by the PANDA toolbox [19].

### Tract-Based Spatial Statistics

The following steps were adopted for the TBSS analysis [17] using FMRIB’s diffusion toolbox (FSL 4.0, http://www.fmrib.ox.ac.uk/fsl). All subjects’ FA images were aligned to a FMRIB-58 FA template in Montreal Neurological Institute (MNI) space using a non-linear registration algorithm. After transformation into MNI space, a mean FA image was created and thinned to generate a mean FA skeleton of the white matter tracts. Each subject’s FA image was then projected onto the skeleton via filling the mean FA skeleton with FA values from the nearest relevant tract center by searching perpendicular to the local skeleton structure for maximum FA value. The registration and projection information derived from the FA analysis were then applied to MD and LDH images of each subject to ensure an exact spatial correspondence of the different parameters.

### Statistical Analysis

A Chi-square test of Pearson and a *t*-test of Student were used to test the group differences in sex and age, respectively. Voxel-wise statistical analysis across subjects on the skeleton space was carried out using a permutation-based inference tool for nonparametric statistic (“randomize”, part of FSL). Group comparisons between schizophrenia patients and healthy controls were performed using a general linear model with age and gender as covariates of no interest. The mean FA skeleton was used as a mask, and the number of permutations was set to 5000. The significance threshold was determined with a *P* < 0.01 (two-tailed) after correcting for family-wise error (FWE) using the threshold-free cluster enhancement (TFCE) [20] option in FSL. Fiber tracts corresponding to the clusters were identified with reference to Johns Hopkins University ICBM-DTI-81 White-Matter Labels provided in FSL toolbox.

## Results

### Demographic and Clinical Characteristics

The demographic and clinical data of the subjects are summarized in Table 1. There were no significant group differences in sex (Chi-square test, χ^2^ = 1.912, *P* = 0.167) and age (two-sample *t* test, *t* = 0.067, *P* = 0.947). 85 patients were receiving atypical antipsychotic medications during the MRI examinations and the other 9 patients had never received any medications. For schizophrenia patients, the mean antipsychotic dosage of chlorpromazine equivalents was 462.5 ± 346.8 mg/d; the mean duration of illness was 123.1 ± 98.6 months; the mean scores of PANSS positive sub-scale and negative sub-scale were 17.1 ± 7.8 and 19.5 ± 8.2.

**Table 1:** Demographic and Clinical Characteristics of the Sample.

Characteristic	Schizophrenia Patients	Healthy controls	*P* value
Number of subjects	94	91	
Age (years)	33.5 ± 8.4	33.5 ± 10.3	0.947
Sex (female/male)	38/56	46/45	0.167
Antipsychotic dosage (mg/d) (chlorpromazine equivalents)	462.5 ± 346.8	NA	
Duration of illness (months)	123.1 ± 98.6	NA	
PANSS			
Positive score	17.1 ± 7.8	NA	
Negative score	19.5 ± 8.2	NA	

The data were shown as the mean values ± standard deviations. Abbreviations: NA, not applicable; PANSS, The Positive and Negative Syndrome Scale.

### Intergroup Differences in FA and MD

**Fig 1:**
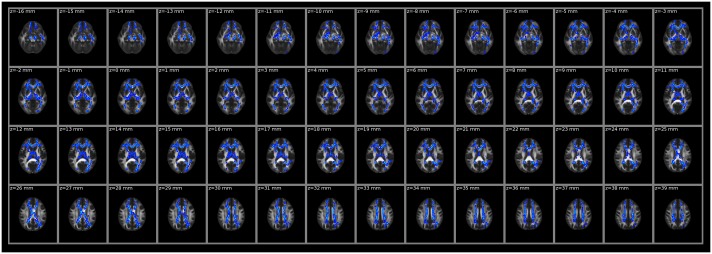
White matter regions with significant differences in FA between patients and controls (*P* < 0.01, two-tailed, FWE corrected). FA, fractional anisotropy.

**Fig 2:**
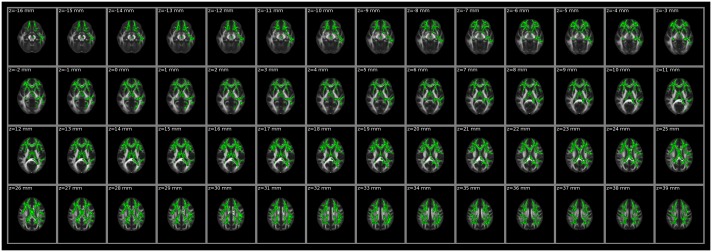
White matter regions with significant differences in MD between patients and controls (*P* < 0.01, two-tailed, FWE corrected). MD, mean diffusivity.

The white matter regions with significant intergroup differences (*P* < 0.01, two-tailed, FWE corrected) in FA and MD are shown in Figs 1 and 2. Schizophrenia patients demonstrated reduced FA and increased MD compared to healthy controls. The distribution patterns of white matter regions with reduced FA and increased MD were similar. The decreased FA and increased MD were mainly located in the corpus callosum, cingulum, internal capsule, fornix and widespread superficial white matter in the frontal, parietal, occipital and temporal lobes.

### Intergroup Differences in LDH

**Fig 3:**
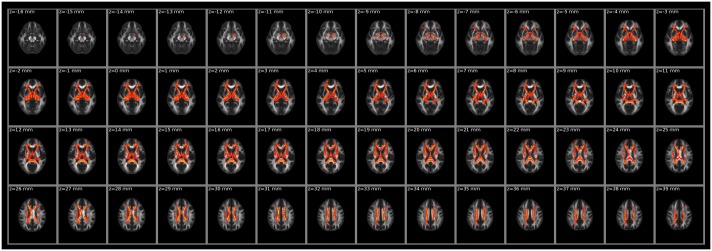
White matter regions with significant differences in LDH between patients and controls (*P* < 0.01, two-tailed, FWE corrected). LDH, local diffusion homogeneity.

The white matter regions with significant intergroup differences (*P* < 0.01, two-tailed, FWE corrected) in LDH are shown in Fig 3. Compared with healthy controls, schizophrenia patients showed decreased LDH mainly in the corpus callosum, cingulum, internal capsule and fornix.

## Discussion

In this study, white matter impairments in schizophrenia patients were detected by both intra-voxel and inter-voxel metrics. We found that schizophrenia patients exhibited reduced FA and increased MD in the corpus callosum, cingulum, internal capsule, fornix and widespread superficial white matter in the frontal, parietal, occipital and temporal lobes. We also found decreased LDH in the corpus callosum, cingulum, internal capsule and fornix in schizophrenia. Our findings suggest that both intra-voxel and inter-voxel diffusion metrics are able to detect impairments in the anisotropic white matter regions, and intra-voxel diffusion metrics could detect additional impairments in the widespread isotropic white matter regions in schizophrenia.

According to a previous study, intra-voxel and inter-voxel metrics may yield discrepant findings even under the same pathological conditions [8]. FA represents a normalized ratio of diffusion directionality, whereas MD quantifies the bulk mobility of water molecules. Accordingly, FA reflects the degree of alignment of cellular structures within fiber tracts and their structural integrity, while MD reflects the overall situation of the whole molecule diffusion level and diffusion resistance. Limited by some conditions, both FA and MD reflect diffusion properties solely within the voxel. However, LDH, which measures the overall coherence of water molecule diffusion within a neighborhood, can reflect the micro-structural coherence of the underlying white matter fibers. Its alterations may represent disruptions of the local coherence of fiber orientations, myelination, diameter or density along the affected white matter tracts. These differences in the type of diffusion metrics may account for the discrepant findings among previous studies. However, more intriguingly, we did not observe any discrepant findings among the metrics of FA, MD and LDH in this study, which is inconsistent with previous findings in other neurological disorders such as Alzheimer’s disease [21] and stroke [8].

The lack of discrepant findings among these three indices in our study may be explained by several potential factors. First, the fact that schizophrenia patients show widespread white matter impairments was previously confirmed by numerous studies, and these white matter impairments included both inter-voxel and intra-voxel micro-structural aberrant white matter. Hence, in the anisotropic white matter regions such as the corpus callosum, cingulum, internal capsule and fornix, schizophrenia patients exhibited both altered inter-voxel and intra-voxel metrics. However, in the isotropic white matter regions such as white matter in the frontal, parietal, occipital and temporal lobes, the local diffusion homogeneity of the fiber tracts may not be affected or may be lightly affected; therefore, FA and MD, rather than LDH, can detect these alterations. Second, the sensitivity of these three indices is different for specific white matter micro-structural properties, which supports the inability of LDH to detect abnormality in fiber tracts in the frontal, parietal, occipital and temporal lobe regions. This study has several limitations that should be addressed in future work. First, we only focused on the capacity of LDH and FA/MD to detect abnormality in the white matter skeleton reconstructed using the TBSS method. These findings should be validated either in white matter regions using an appropriate atlas or in white matter fiber tracts reconstructed by tractography in future studies. Second, our study lacked correlation analyses between imaging parameters and clinical data. The primary purpose of this study was to test the feasibility of LDH in schizophrenia research. Thus, future studies that evaluate correlations of DTI parameters with clinical characteristics are needed.

In conclusion, our findings suggest that both intra-voxel and inter-voxel diffusion metrics are able to detect impairments in the anisotropic white matter regions, and intra-voxel diffusion metrics could detect additional impairments in the widespread isotropic white matter regions in schizophrenia. Thus, the combination of two types of diffusion metrics may provide complementary information for understanding the underlying pathological changes in schizophrenia.
